# 

**Published:** 1917-03-24

**Authors:** 


### THE HOSPITAL

**The Modern Newspaper of Administrative Medicine and Institutional Life.**

Telegraphic Address: OSPEDALE, RAND, LONDON. Telephone Number: 2734 GERRARD.

No. 1607, Vol. LXI. | Saturday, March 24th, 1917 | PRICE ONE PENNY

